# Monogenic hypertriglyceridemia and recurrent pancreatitis in a homozygous carrier of a rare *APOA5* mutation: a case report

**DOI:** 10.1186/s13256-024-04532-0

**Published:** 2024-06-14

**Authors:** Umidakhon Makhmudova, P. Christian Schulze, Stefan Lorkowski, Winfried März, J.-A. Geiling, Oliver Weingärtner

**Affiliations:** 1 Deutsches Herzzentrum der Charité, Hindenburgdamm 30, 12203 Berlin, Germany; 2 Friede Springer Cardiovascular Prevention Center @Charité, Hindenburgdamm 30, 12203 Berlin, Germany; 3 Charité-Universitätsmedizin Berlin, Freie Universität Berlin and Humboldt-Universität zu Berlin, Klinik/Centrum, Charitéplatz 1, 10117 Berlin, Germany; 4 Klinik für Innere Medizin I, Universitätsklinikum Jena, Am Klinikum 1, 07743 Jena, Germany; 5 Institute of Nutritional Sciences, Friedrich Schiller University Jena, Jena, Germany; 6 Competence Cluster for Nutrition and Cardiovascular Health (nutriCARD), Halle-Jena-Leipzig, Germany; 7 SYNLAB Academy, SYNLAB Holding Deutschland GmbH Mannheim and Augsburg GmbH, Mannheim, Germany

**Keywords:** Monogenic hypertriglyceridemia, *APOA5*, Acute pancreatitis, Volanesorsen, Case report

## Abstract

**Background:**

Homozygous mutations in the *APOA5* gene constitute a rare cause of monogenic hypertriglyceridemia, or familial chylomicronemia syndrome (FCS). We searched PubMed and identified 16 cases of homozygous mutations in the *APOA5* gene. Severe hypertriglyceridemia related to monogenic mutations in triglyceride-regulating genes can cause recurrent acute pancreatitis. Standard therapeutic approaches for managing this condition typically include dietary interventions, fibrates, and omega-3-fatty acids. A novel therapeutic approach, antisense oligonucleotide volanesorsen is approved for use in patients with FCS.

**Case presentation:**

We report a case of a 25-years old Afghani male presenting with acute pancreatitis due to severe hypertriglyceridemia up to 29.8 mmol/L caused by homozygosity in *APOA5* (c.427delC, p.Arg143Alafs*57). A low-fat diet enriched with medium-chain TG (MCT) oil and fibrate therapy did not prevent recurrent relapses, and volanesorsen was initiated. Volanesorsen resulted in almost normalized triglyceride levels. No further relapses of acute pancreatitis occurred. Patient reported an improve life quality due to alleviated chronic abdominal pain and headaches.

**Conclusions:**

Our case reports a rare yet potentially life-threatening condition—monogenic hypertriglyceridemia-induced acute pancreatitis. The implementation of the antisense drug volanesorsen resulted in improved triglyceride levels, alleviated symptoms, and enhanced the quality of life.

**Supplementary Information:**

The online version contains supplementary material available at 10.1186/s13256-024-04532-0.

## Background

Although there is no commonly accepted triglyceride (TG) cut-off value, hypertriglyceridemia (HTG) is usually defined as serum TG above 1.7 mmol/L, measured in the non-fasting state. TG levels higher than 10 mmol/L are considered severe HTG and are associated with an increased risk of acute pancreatitis [1–3].

Primary HTG can be monogenic or polygenic. According to Hegele *et al*. [4], 28% of the general population have TG levels of 2–10 mmol/L due to polygenic HTG. Polygenic HTG mainly occurs due to heterozygous mutations in lipoprotein lipase *(LPL)*, apolipoprotein A5 (*APOA5*), glucokinase regulator (GCKR), apolipoprotein B (*APOB)*, lipase maturation factor *(LMF1)*, glycosylphosphatidylinositol-anchored high-density lipoprotein-binding protein 1 (*GPIHBP1*), cAMP response element-binding protein 3-like 3 (*CREB3L3*), apolipoprotein C2 (*APOC2*), apolipoprotein APOE (*APOE*) and other small-effect variants. The prevalence of monogenic HTG (also coined FCS or primary hyperlipoproteinemia type I or V according to Fredrickson) in the population is 0.1–2% [2]. Monogenic HTG occurs due to homozygous or compound heterozygous mutations in *LPL*, *APOA5*, *APOC2*, *LMF1*, *GPIHBP1* or glycerol-3-phosphate dehydrogenase (*GPDH*) [5]. In most cases, monogenic HTG occurs due to *LPL* mutations [2], while *APOA5* homozygous mutations are rare.

## Case presentation

### Clinical presentation

A 25-years-old male of Afghani origin with a history of recurrent acute pancreatitis was referred to the intensive care unit of the Jena University Hospital in November 2019.

Abdominal pain debuted two years ago when the patient was 23 years old. The family history revealed that the patient’s brother and sister similarly suffer from abdominal pain and have been hospitalized several times. His niece died at the age of three due to acute pancreatitis. The patient’s whole family resides in Afghanistan, so neither clinical nor genetic investigation of relatives was possible in our clinic.

On physical examination, the patient’s BMI was 23.4 kg/m^2^, blood pressure, and heart rate were 146/83 mm Hg and 95/min, respectively. He showed no xanthomas but did have lipemia retinalis on retinal examination. Examination of the abdomen revealed pain in the upper left area and an enlarged spleen. No other abnormalities were observed.

### Investigations

#### Laboratory findings

TG levels were at 29 mmol/L, and low-density lipoprotein (LDL) and high-density lipoprotein cholesterol (HDL) cholesterol were within reference ranges. According to the previous records, TGs were as high as 82 mmol/L in the past. Inflammation markers (C-reactive protein and white blood cells) were remarkably elevated. There were no clinical symptoms or laboratory indicators of secondary (pancreoprivic) diabetes mellitus (HbA1c 5.1%, blood glucose 5.8 mmol/L). Other laboratory parameters are shown in Table 1.

**Table 1:** Laboratory parameters upon admission

Analyte	Result	Normal range
Triglycerides	29.75	< 1.7 mmol/l
LDL-C	0.92	< 3.35 mmol/l
HDL-C	− 0.55	> 1.03 mmol/l
Total cholesterol	3.03	< 5.2 mmol/l
CRP	+ 140.5	< 7.5 mg/dl
ALT	0.47	< 0.58 µmol/l
AST	0.22	< 0.74 µmol/l
Bilirubin (direct)	30	< 3 µmol/l
Gamma GT	0.27	< 0.92 µmol/l
Lipase	8.9	< 1.33 µmol/l*s
Amylase	n.a. (lipaemic)	< 1.68 µmol/l*s
RBC	5.9	4.5–5.9 Tpt/l
WBC	19.6	4.4–11.3 Gpt/l
Platelets	208	150–360 Gpt/l

*LDL* low-density cholesterol, *HDL* high-density cholesterol, *CRP* C-reactive protein, *ALT* alanin aminotransferase, *AST* aspartate aminotransferase, *Gamma GT* Gamma guanyltransferase, *RBC* red blood cells, *WBC* white blood cells

#### Imaging

Abdominal computer tomography demonstrated edematous pancreatitis, most prominently within the corpus pancreaticus with surrounding fat tissue fibrosis and splenomegaly. There were no signs of choledocholithiasis.

#### Genetic study

A panel screening for HTG-related genes (Additional file 1) was performed. The sequencing was performed using the next-generation sequencing on Illumina-Sequencer (NextSeq500/NovaSeq6000) with a > 98% coverage of regions of interest. The analysis revealed homozygosity for a frameshift mutation of *APOA5* (c427delC, p.Arg143Alafs*57) with a minor allele frequency of 0.006%. This mutation causes an alteration in the translational reading frame and results in a premature stop of protein synthesis due to the introduction of a stop codon at position 57. The patient was also a homozygous carrier of haplotype APOA5*2.

### Treatment

**Fig. 1:**
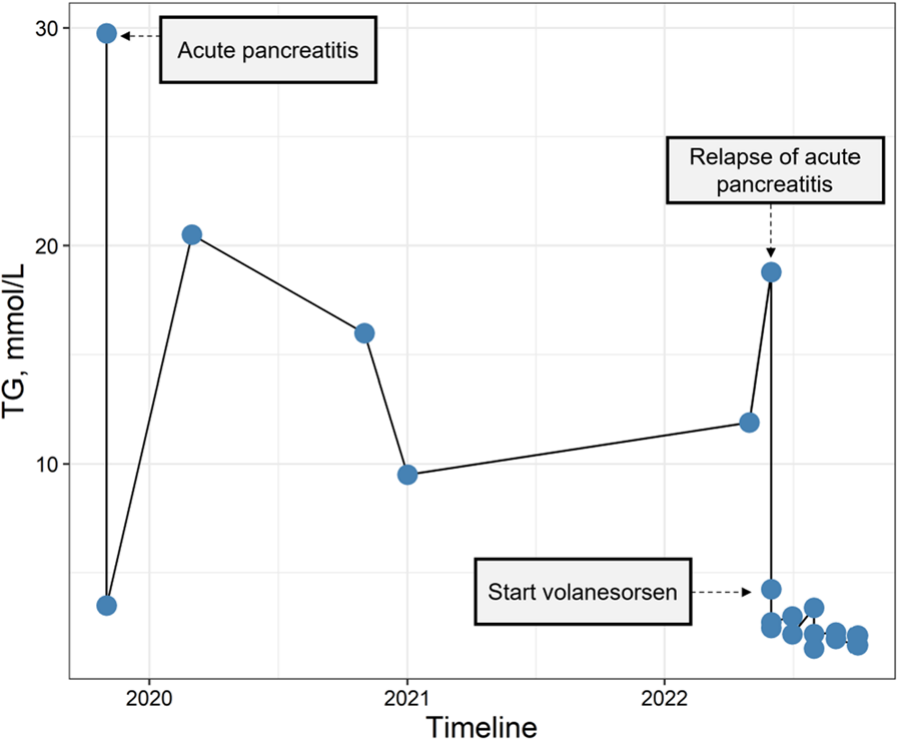
Triglyceride levels dynamics. After the therapy initiation, triglyceride levels improved and remained between 8 and 11 mmol/L, until May 2022, when a new episode of acute pancreatitis occurred. Volanesorsen was started shortly afterwards and results in the normalization and stabilization of triglyceride levels

**Fig. 2:**
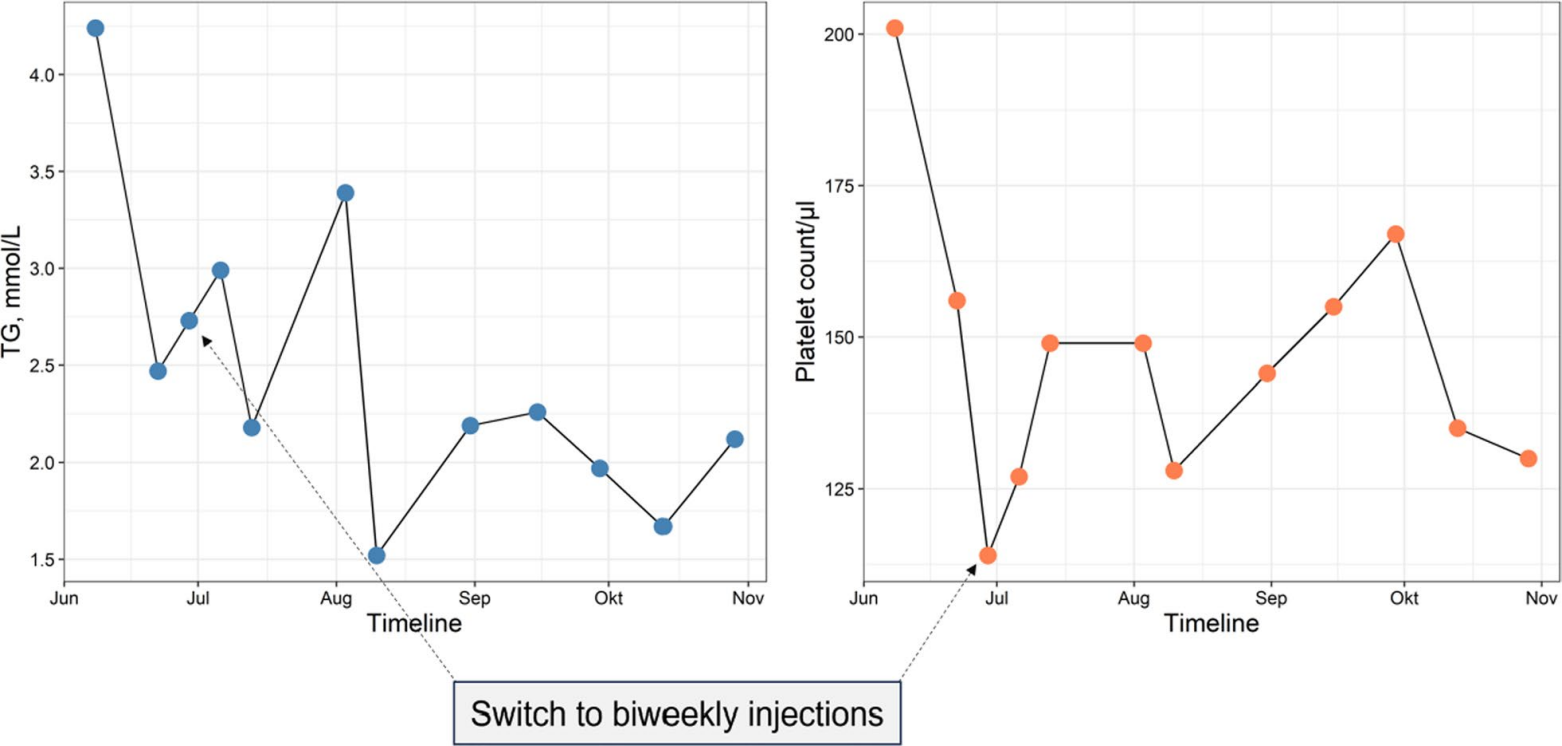
triglyceride levels and platelet count after initiation of volanesorsen therapy. Following the start of volanesorsen therapy, platelet count and triglyceride levels were monitored. Volanesorsen treatment commenced shortly after a hospitalization due to acute pancreatitis, during which triglyceride levels peaked at approximately 18 mmol/L. Prior to discharge, triglyceride levels returned to normal, and volanesorsen therapy was initiated. Three weeks into the treatment, the platelet count decreased to 114,000/µl, prompting a switch to biweekly volanesorsen administration. This modified regimen led to the normalization of platelet count, maintaining stability at over 125,000/µl, alongside consistent triglyceride levels

Acute pancreatitis was treated with aggressive fluid resuscitation and therapeutic plasma exchange (Spectra Optia, Terumo BCT, Inc. Lakewood USA). The patient was then put on a low-fat diet and a combination of ezetimibe (10 mg daily) and fenofibrate (160 mg micronized daily). Shortly after the discharge, another episode of acute pancreatitis occurred. The patient was prescribed omega-3 fatty acids; however, the inability to obtain reimbursement for omega-3 fatty acids in Germany hindered their usage. The patient was regularly followed up at our outpatient clinic. A satisfactory range of TG, between 9.2 and 11.2 mmol/L (Fig. 1), was maintained through a combination of stringent dietary measures and consistent intake of fibrates. After two years, another episode of pancreatitis occurred, with TG elevation up to 18.8 mmol/L. The patient was started on weekly injections of volanesorsen. On this regimen, TG levels were stably under 4 mmol/L (Fig. 2). The platelet count decreased from 201,000 to 114,000/µL. Therefore, according to recommendations, we switched to biweekly administrations. As a result, the platelet count stabilized (at ~ 150,000/µL). The patient had no episodes of bleeding. Volanesorsen therapy was continued with regular assessment of platelet count.

## Discussion

We describe a case of recurrent acute pancreatitis due to homozygosity in APOA5 gene (c427delC, p.Arg143Alafs*57).

The patient had a history of severe HTG and had suffered from a series of pancreatitis episodes. A low-fat diet enriched with MCT oil and fibrate therapy was insufficient to prevent recurrent pancreatitis, and the patient was put on volanesorsen—an antisense oligonucleotide against *APOC3* mRNA. Volanesorsen reduced TG levels, though causing transient mild thrombocytopenia, which was managed by increasing the interval between injections.

The pathogenesis of hypertriglyceridemia-induced pancreatitis (HTGP) is linked to the accumulation of free fatty acids (FFA) and activation of inflammatory response. Excess TGs are hydrolyzed by pancreatic lipase to FFA, which leads to acinar cell injury and pancreatic capillary ischemia [5]. Free fatty acids additionally increase proinflammatory triggers like TNF-a, interleukin-6, and interleukin-10, and these inflammatory cytokines enhance hepatic triglyceride production [6]. Another important pathogenetic mechanism in the development of HTGP involves disturbances in the pancreatic microcirculation, caused by the imbalance of vasoconstrictors and vasodilators [6]. In addition, hyperviscous blood leads to capillary obstruction in the pancreas [7]. Severe HTG is responsible for ~ 10% of incidents of acute pancreatitis, ranking as the third most prevalent underlying cause [6, 8, 9].

This mutation was first reported by Thèrilaut *et al*. [10]. It most probably leads to a truncated protein, which cannot interact with LPL, so LPL is unable to hydrolyze and reduce serum TG levels [10]. The patient described by Thèrilaut *et al*. was referred due to abdominal bloating but had no abdominal pain or pancreatitis. He also appeared to have a coronary artery anomaly, prolonged QT time, and a positive family history of cardiovascular disease. Medical management included diet and fibrates. However, the treatment had no significant impact on blood TG levels.

At variance, our patient had been hospitalized several times due to acute pancreatitis therapeutic plasma exchange and intensive fluid resuscitation improved the clinic in the acute situation. Following a low-fat diet and a combination treatment of fenofibrate and ezetimibe, the patient attained TG levels within the range of 9.2–11.2 mmol/l. The patient has remained relapse-free for more than a year. However, he suffered from mild chronic abdominal pain and headaches. After another recurrent episode of acute pancreatitis with TG levels ~ 18 mmol/L, the patient was started on volanesorsen therapy. TG levels were subsequently normalized. After the fourth injection, the platelet count decreased to 114,000/µl, so we switched to biweekly instead of weekly injections. As a result, chronic symptoms (abdominal pain, headaches) have resolved, and the patient has remained relapse-free.

To summarize, this case report demonstrates a rare mutation in the *APOA5* gene, which led to severe HTG, causing recurrent acute pancreatitis episodes in a young male. A notable limitation of this case is the absence of omega-3 fatty acids treatment, which was unavailable to our patient due to reimbursement challenges. Additionally, while we did not conduct lipoprotein electrophoresis, given the genetic analysis revealed a homozygous mutation deemed the most likely cause of severe HTG, lipoprotein electrophoresis could still be useful in individuals with multiple pathogenic variants to differentiate between familial chylomicronemia syndrome (FCS) and multifactorial chylomicronemia.

### Literature review

#### *APOA5* function

*APOA5* gene was first described only around 20 years ago and is the newest member of the *APOA* class genes [11]. Although its protein concentration in serum is very low, it has a substantial effect on reducing TG levels [12, 13]. *APOA5* is synthesized in the liver and secreted with very low-density lipoprotein (VLDL) particles. *APOA5*-knockout mice have four-fold higher TG plasma levels in comparison to wild-type animals [8]. Consistently, expression of *APOA5* in transgenic mice results in a 50–70% decrease in plasma TG levels [14]. Two mechanisms explain the TG-regulating role of *APOA5*: (i) *APOA5* modulates the catabolism of TGs by stimulating LPL-mediated TG hydrolysis due to interaction with GPIHBP1 [10, 15, 16]; (ii) it inhibits the production of VLDL, which is a significant carrier of TGs [17]. Generally, more than 400 genetic variants of *APOA5* are known to date [7]. Severe HTG is linked to only a few *APOA5* mutations.

#### *APOA5* homozygous mutations: previous cases

*APOA5* homozygous mutations are rare. The first case of *APOA5* complete deficiency was identified by sequencing *APOA5* in 10 hypertriglceridemic subjects [18]. We searched PubMed for cases of homozygous *APOA5* mutations with keywords “APOA5” OR “APOA5 mutation*” OR “APOA5 homozyg*”, and identified 16 cases of *APOA5* homozygosity (Table 2). We also identified one case of loss-of-function heterozygosity, resulting in severe HTG, and one case of compound heterozygosity. The most common mutation was Q97X (c.289 C > T), which was reported in four subjects.

**Table 2:** Reported cases of homozygous *APOA5* mutation (demographics, clinical presentation, TG levels, therapy)

Publication/mutation	Onset *	Sex	Acute pancreatitis	Other symptoms/Signs	TG, mmol/L	Therapy	TG after therapy, mmol/L
Oliva 2005 [18] *c.433 C* > *T*	5	M	Yes	Planar xathomas, eruptive cutaneous xanthomas, mild hepato-splenomegaly	> 50	Low-fat diet, omega-3-fatty acids	< 8
Marçais 2005 [32]^(p5)^ *Q139X*	34	M	Yes	Myocardial infarction	> 40	Diet, omega-3-fatty acids, fibrate	6.5–60
Henneman 2007 [40] *c.161* + *5G* > *C*	31	F	No	No abnormalities	19.4	Low-fat diet Discontinuation of oral anticontaceptiva	6–13.3 1.8
Oliva 2008 [33] *c.289 C* > *T* *(Q97X)*	2	M	No	Eruptive xanthomas, no hepatosplenomegaly	9.65	Low-fat diet, Omega-3-fatty acids	2.4–12.0
Charriere 2009 [34] *c.289 C* > *T* *(Q97X)*	25	M	Not reported	Not reported	12.84	Low-fat diet	Normal to moderately elevated
Okubo 2009 [37] *c.49* + *1 g* > *a*	35	M	Yes	No diabetes	20.5	Not reported	Not reported
Dussaillant 2012 [26] *c.289C* > *T* *(Q97X)*	22	F	Yes	Not reported	112	Fibrate/nicotinic acid	Not reported
Dussaillant 2012 [26] *c.289C* > *T* *(Q97X)*	30	F	Not reported	Pre-diabetes, overweight	116	Omega-3-fatty acids	Not reported
Mendoza-Barbera 2013 [36] *c.757 T* > *C*	5	F	No	Discovered during a routine check-up, no abnormalities	15.3	Not reported	Not reported
Mendoza-Barbera 2013 [36] *c.289C* > *T* *(compound heterozygosity)*	4	F	Not reported	Tuberous xanthoma	19.9	Not reported	Not reported
Albers 2014 [19] *c.16_39del*	11 months	M	Yes	Not reported (myocardial infarctions in the family)	25.2	Weaning and switch to regular formula-based diet	2.27
Hooper 2014 [35] *c.823C* > *T*	16	F	Yes	Diabetes, hypertension	28.5	Low-fat diet, omega-3 fatty acids	Not reported
Hooper 2014 [35] *c.823C* > *T*	70	F	Yes	Obesity, diabetes, hepatosplenomegaly	94	Low-fat diet, fibrate, omega-3 fatty acids, statin, therapeutic plasma exchange	Not reported
Thériault 2016 [10] *c.G425del-C*	12	M	Abdominal bloating	Xanthomas, hemangioma of the spleen, coronary artery anomaly	8.93 (up to 35 in the past)	Diet, fibrate	7.95
Buonuomo 2017 [41] *c.883C* > *T*	8	M	No	No abnormalities	11.65	Hypolipidemic diet Omega-3 fatty acid DHA-rich oil pearls	3.8–7.4
Vasiluev 2022 [38] *c.579_592delATACGCCGAGAGCC*	4	M	Yes	Hepatosplenomegaly, xanthomas	55	Low-fat diet, iron, ursodeoxycholic acid, antihistamines, interferon, intestinal microflora stabilizers	6.3
Loh 2022 [39] *c.553G* > *T*	38	M	No	Subarachnoid haemorrhage No eruptive xanthomas, no lipemia retinalis, no diabetes	52.4	Intravenous dextrose-insulin infusion, fenofibrate, and atorvastatin	< 10
Isaac 2023 [20] *c.(50–1)_(161* + *1_162-1)* *(loss-of-function heterozygous)*	21	F	Yes	No eruptive xanthomas, no lipemia retinalis, no overweight, no diabetes, no hepatosplenomegaly	38.6 at first episode of acute pancreatitis, maximal value: 130,8	Fibrates, statins, insulin, omega-3 free fatty acids, exercise, diet (low-fat/carbohydrate);	No change
						Volanesorsen	< 2.26
Present case *c.G425del-C*	25	M	Yes	Lipemia retinalis	29.8	Fibrate, ezetimibe, diet, MCT oil	8–11
						Volanesorsen	< 4

To maintain consistency, TG levels originally measured in mg/dL were converted to mmol/L using the formula: TG (mmol/L) = TG (mg/dL)/88.57

*TG* triglyceride

The clinical presentations of these cases varied, ranging from individuals with no apparent symptoms (elevated triglycerides identified during routine examinations) to cases of acute pancreatitis. The earliest age at the debut of acute pancreatitis was as young as 11 months of age [19]. Triglyceride levels varied as well, ranging from a minimum of ~ 9.65 mmol/L to a maximum of 130.8 mmol/L mmol/L (median 29 mmol/L). Treatment approaches for these patients typically involved a combination of a low-fat diet, fibrates, and omega-fatty acid supplementation, yielding various outcomes. The ASO volanesorsen treatment was administered in only one instance, specifically in the case of loss-of-function heterozygosity resulting in severe HTG (TG levels up to 108 mmol/L) and causing acute pancreatitis [20]. The patient was treated with volanesorsen, which led to consistent and lasting normalization of TG levels to below 2.26 mmol/L.

#### Treatment of monogenic hypertriglyceridemia (FCS)

##### Diet

A low-fat diet is a cornerstone of the treatment. It is recommended to restrict daily fat consumption to 15–20 g or restrict total daily fat intake to 15% of daily calorie intake or individualize fat intake to assure the daily essential fatty acid (EFA) needs of 2–4% daily calorie intake of linolenic acid and alpha-linolenic acid [21, 22]. Distributing the portions over the day is necessary to avoid a TG increase. Even a small deviation in the diet results in a significant increase in the risk for acute pancreatitis [22].

MCTs are recommended in FCS because they are not transported via chylomicrons. To ensure sufficient protein intake, it is also recommended to consume low-fat or fat-free protein products. To note, unsaturated fat (fish, nuts, seeds) is also metabolized by the LPL pathway and, therefore, is not recommended in patients with FCS. Patients should avoid simple carbohydrates, such as candy, sugary drinks, syrups, and fruit juice concentrate [22].

##### Fibrates

Fibrates can lower TG levels by up to 50% by inhibiting apoC3 expression and VLDL production in the liver via the PPAR-a (Peroxisome proliferator-activated receptors-a) pathway. The efficacy of fibrates in clinical trials was demonstrated in patients with TG < 11.3 mmol/L. One retrospective study reported fibrate use in a cohort with severe HTG (mean TG 35.04 mmol/L); 15.8% of them had episodes of acute pancreatitis [23]. In this study, most patients received fibrate monotherapy or a fibrate in combination with a statin, and TGs were reduced from 35 to 8 mmol/L. In three patients with LPL deficiency, fibrates reduced TG levels from 23–34 to < 2.8 mmol/L. However, there is no sufficient evidence that fibrates efficiently reduce TG levels in subjects with *APOA5* mutation. Moreover, several studies have indicated an increased risk of pancreatitis associated with fibrates [24, 25]. This is likely attributable to an augmentation in cholesterol content within the bile, leading to the formation of gallstones.

##### Niacin

Niacin inhibits hormone-sensitive TG lipase. This leads to decreased lipolysis and free fatty acid release from adipose tissue. It reduces TG by 5 to 35%. However, it is not widely used due to substantial side effects (hepatotoxicity, impaired glucose tolerance, hyperuricemia, flush). Dussailant *et al*. reported successful use of niacin in a patient with *APOA5* Q97X mutation [26].

##### Omega-3-fatty acids

Omega-3 fatty acids inhibit VLDL production, reduce chylomicron size, increase systemic lipolysis, and promote chylomicron plasma clearance. Omega-3 acid ethyl esters lower TG concentrations by 20–50% [27] and may reduce the recurrence and complications rate of acute pancreatitis [28]. Current ESC/EAS Guidelines on dyslipidemia recommend initiating fibrate therapy with omega-3 fatty acids (2–4 g/day) [1]. Although initially prescribed, it was not possible to treat our patient with omega-3 acid ethyl ester due to health insurance issues: starting from April 2020, omega-3 acid ethyl ester is not reimbursed anymore in Germany.

##### ApoC3 inhibition

ApoC3 is synthesized in hepatocytes and enterocytes. ApoC3 inhibits the LPL-mediated lipolysis of chylomicrons and VLDL and disrupts the hepatic clearance of remnants. As a result, ApoC3 knockout mice are hypotriglyceridemic.

Volanesorsen, a second-generation antisense oligonucleotide (ASO), targets APOC3 mRNA, effectively reducing triglyceride (TG) levels in the bloodstream. It received approval from the European Medicinal Agency in 2019 for the treatment of adult patients with FCS [29].

In the phase 3 randomized controlled trial APPROACH, volanesorsen showed a 77% reduction in TG levels among patients with FCS [30]. The most common side effects observed were reactions at the injection site and a decrease in platelet count, with 7/33 patients experiencing platelet counts below 50,000/µl. A similar phase 3 study, COMPASS, yielded comparable results, demonstrating a 71% reduction in mean TG levels [31]. Throughout the study duration, there were five cases of acute pancreatitis, all occurring in three patients from the placebo group. Volanesorsen was generally well-tolerated, with injection site reactions being the most common side effect. Thrombocytopenia (< 50,000/µl) was reported in only one out of 76 patients.

## Conclusions

Monogenic mutations causing HTG are rare. We here report a case of a young male in his 20 s with a frameshift mutation of *APOA5* and severe HTG, who suffered from recurrent acute pancreatitis. Diet and therapy with fibrate and ezetimibe combination achieved TG levels of 9–11 mmol/L. Due to recurrent episodes of acute pancreatitis, the patient was started on volanesorsen. This resulted in the normalization of TG levels, prevention of acute pancreatitis relapses and improvement of quality of life. A mild thrombocytopenia was managed by strict control of platelet count and switching to biweekly injections of the drug.

### Supplementary Information

**Additional file 1.** List of analyzed genes.

## Data Availability

The data used and/or analyzed during the current study are available in anonymized form from the corresponding author on reasonable request.
