# Supplementary material for: Monogenic hypertriglyceridemia and recurrent pancreatitis in a homozygous carrier of a rare APOA5 mutation: a case report
Source: J Med Case Rep. 2024 Jun 14;18:278. doi: 10.1186/s13256-024-04532-0 (PMC11177521; doi:10.1186/s13256-024-04532-0)
Supplement: Supplementary file 1 — Additional file 1. List of analyzed genes. [file 13256_2024_4532_MOESM1_ESM.docx]

**Additional material.**

List of analyzed genes (NGS):

- APOA5 - Apolipoprotein A-V
- APOB - Apolipoprotein B
- APOC2 - Apolipoprotein C-II
- APOC3 - Apolipoprotein C-III
- APOE - Apolipoprotein E
- GCKR - Glucokinase Regulatory Protein
- GK - Glycogen Kinase
- CPD1 - Carboxypeptidase D
- CPIHBP1 - Lipase Cofactor (Colipase) Heparin-Binding Protein 1
- LIPC - Hepatic Lipase
- LMF1 - Lipase Maturation Factor 1
- LPL - Lipoprotein Lipase
- USF1 - Upstream Transcription Factor 1
